# From Tyrosine Kinases to Tyrosine Phosphatases: New Therapeutic Targets in Cancers and Beyond

**DOI:** 10.3390/pharmaceutics16070888

**Published:** 2024-07-01

**Authors:** Yu Zhou, Zhimeng Yao, Yusheng Lin, Hao Zhang

**Affiliations:** 1 State Key Laboratory of Bioactive Molecules and Druggability Assessment, MOE Key Laboratory of Tumor Molecular Biology, and Institute of Precision Cancer Medicine and Pathology, School of Medicine, Jinan University, Guangzhou 510632, China; yuzloveflower@163.com (Y.Z.); crcjnu@163.com (Z.Y.); easonlin0423@126.com (Y.L.); 2 Department of Urology Surgery, The First Affiliated Hospital of Jinan University, Jinan University, Guangzhou 510660, China; 3 Department of Thoracic Surgery, The First Affiliated Hospital of Jinan University, Jinan University, Guangzhou 510660, China; 4 Department of Pathology, Gongli Hospital of Shanghai Pudong New Area, Shanghai 200135, China; 5 Zhuhai Institute of Jinan University, Zhuhai 511436, China

**Keywords:** protein tyrosine phosphatases, protein tyrosine kinases, tumor suppressor, immune responses, nervous system, drug resistance

## Abstract

Protein tyrosine kinases (PTKs) and protein tyrosine phosphatases (PTPs) regulate the level of tyrosine phosphorylation in proteins. PTKs are key enzymes that catalyze the transfer of an ATP phosphoric acid to a tyrosine residue on target protein substrates. Protein tyrosine phosphatases (PTPs) are responsible for the dephosphorylation of tyrosine residues and play a role in countering PTK overactivity. As widespread oncogenes, PTKs were once considered to be promising targets for therapy. However, tyrosine kinase inhibitors (TKIs) now face a number of challenges, including drug resistance and toxic side effects. Treatment strategies now need to be developed from a new perspective. In this review, we assess the current state of TKIs and highlight the role of PTPs in cancer and other diseases. With the advances of allosteric inhibition and the development of multiple alternative proprietary drug strategies, the reputation of PTPs as “undruggable” targets has been overturned, and they are now considered viable therapeutic targets. We also discuss the strategies and prospects of PTP-targeted therapy, as well as its future development.

## 1. Introduction

**Figure 1:**
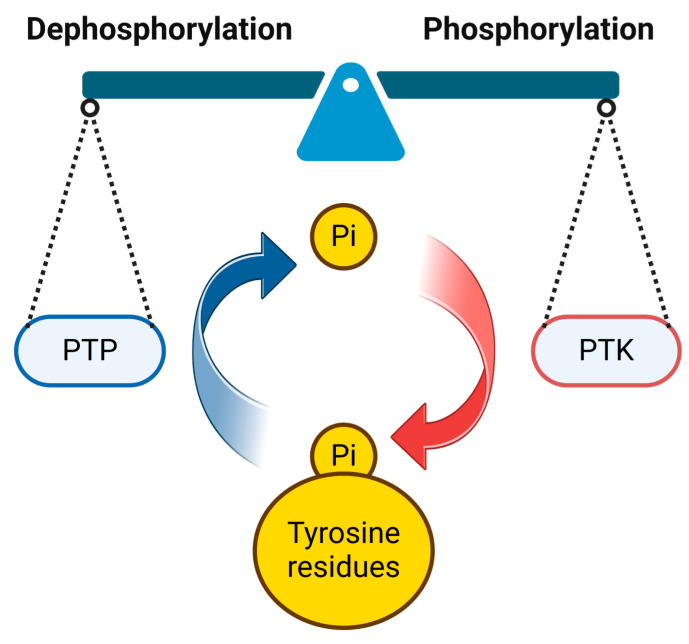
Balance of tyrosine residue phosphorylation status in target proteins and their regulation by protein tyroTsine phosphatases (PTPs) and protein tyrosine kinases (PTKs).

Protein tyrosine phosphorylation—a pivotal post-translational modification—is orchestrated by protein tyrosine kinases (PTKs) and reversed by protein tyrosine phosphatases (PTPs) and plays a critical role in cellular signaling (Figure 1). Significantly, many human diseases can result from upsetting the equilibrium of this phosphorylation balance [1,2,3]. So far, blocking carcinogenic tyrosine phosphorylation has been a promising target for tyrosine kinase inhibitors (TKIs) [4,5]. In clinical practice, however, very few kinase inhibitors have a long-lasting therapeutic effect on cancer [6]. The majority of kinase inhibitors cause treatment resistance, aberrant signaling pathway activation, and rapid compensatory signaling [6,7,8,9]. The current generation of TKIs is restricted in scope, primarily inhibiting the de novo phosphorylation of tyrosine residues [10], thereby necessitating the exploration of novel therapeutic strategies.

The dysregulation of PTP-associated tyrosine phosphorylation has been implicated in many human diseases, including malignancies, neurological diseases, immune disorders, cardiovascular and metabolic diseases [11,12,13,14,15,16,17,18,19,20,21,22]. Like kinases, PTPs exhibit dual functionality, serving as either tumor suppressors or oncogenes. This duality enables PTPs to influence tumor growth, either by directly modulating key tumor-associated kinases or by impacting numerous downstream signaling pathways [3,23,24,25,26]. This provides us with at least two approaches to treating cancer: reactivating suppressed tumor suppressor phosphatases or blocking carcinogenic phosphatases. Understanding the structure and regulatory mechanisms of phosphatases more thoroughly is necessary, given the recent success in selectively targeting them.

The development of PTP-targeting drugs has, however, been fraught with challenges, largely due to the highly conserved nature of the PTP active site [27,28]. The field is advancing, with novel approaches such as irreversible and non-competitive inhibitors, along with the conventional reversible competitive methods, gaining momentum [27,29,30,31,32,33,34]. Notably, research into allosteric small-molecule inhibitors, which bypass charged PTP active sites, holds significant promise [30,32,35]. Furthermore, PTP drugs based on nucleic acids that target activation or inhibition, as well as substances that stabilize protein components or protein–protein interactions, are currently being developed [36,37,38,39,40,41].

## 2. Status of TK Inhibitors

### 2.1. Advantages of TKIs

Tyrosine kinases (TKs) catalyze the transfer of ATP phosphate groups to the tyrosine residues of target proteins [42]. TKs are subclassified into receptor tyrosine kinases (RTKs) and non-receptor tyrosine kinases (nRTKs) [43,44]. Tumor initiation frequently involves the overexpression and mutation of TKs, positioning the inhibition of these kinases as a crucial therapeutic strategy [45]. A tyrosine kinase inhibitor (TKI) is often a homolog of adenosine triphosphate (ATP). TKIs are split into at least two groups: ones that target the ATP site and ones that do not. The former inhibits the growth and proliferation of cancer cells by competitively occupying the ATP binding site of tyrosine kinases and blocking the tyrosine-kinase-mediated signaling pathway. Salient examples of this include the potent antitumor efficacy of TKIs when targeting tyrosine kinases, such as the epidermal growth factor receptor (EGFR)/phosphatidylinositol 3 kinase (PI3K) and mitogen-activated protein kinase (MAPK) signaling pathways [12,46,47]. The emergence of TKIs, such as sorafenib for hepatocellular carcinoma and afatinib for lung adenocarcinoma, was considered a new page in cancer research and clinical practice [48,49]. As of February 2024, the FDA has approved 80 small-molecule protein kinase inhibitors—a significant increase from the 23 approved in 2018—reflecting the rapid expansion of this therapeutic area [4]. Most TKIs (such as sorafenib, imatinib, and masitinib) can be administered orally, which has important implications for patient compliance, safety, and cost-effectiveness. TKIs must be designed with full consideration of their aim, which is to bind to already defined binding targets to inhibit specific tyrosine kinase activity. TKIs offer significant advantages over conventional chemotherapy medications in terms of efficiency, toxicity, and specificity [5]. These superior qualities facilitate a simplified yet detailed pharmacokinetic/pharmacodynamic (PK/PD) modeling approach, where drug concentration is directly linked to target engagement and downstream effects [50,51,52]. Such traits provide substantial benefits in PK/PD modeling studies focused on TKIs, as they can guide dosage optimization and strategy enhancement effectively.

### 2.2. Challenges for TKIs 

#### 2.2.1. Drug Resistance

Cancer recurrence due to drug resistance is an important issue in cancer management [53]. During TKI therapy, drug resistance can be divided into primary (intrinsic) resistance and secondary (acquired) resistance. Acquired resistance may originate from selection against genetic or epigenetic alterations in cancer cells [54,55,56]. The main mechanisms include the following:

Altered target gene modification: Cancer cells may undergo altered modification of the target gene itself, or the effects may be downstream of the oncogenic pathway in which it participates. Such alterations can prevent TKIs from binding adequately to target interactions, and insufficient inhibition of TKs leads to decreased drug efficacy. For example, in non-small-cell lung cancer (NSCLC), mutations in the structural domain of the epidermal growth factor receptor (EGFR) kinase render patients resistant to EGFR inhibitors such as gefitinib and erlotinib [57,58]. Tumor cells with mutations in the EGFR or Kirsten rat sarcoma viral oncogene (KRAS) also demonstrate resistance to TKIs [59,60].

Activation of bypass signaling: When TKIs function to inhibit signaling pathway transduction by binding to a specific target, cancer cells may activate bypass signaling, a new mode of tumor progression that is not dependent on that specific target. This bypass signaling can bypass the inhibition of the target pathway or activate compensatory feedback loops, thereby restoring tumor growth. TKIs can induce the prolonged inhibition of specific pathways, which may induce tumor phosphorylation-dependent signaling rearrangements. For example, upon inhibition of TKIs, tumor cells show amplification of alternative receptor tyrosine kinases (RTKs) that are not targeted by the drugs [56]. Mesenchymal–epithelial transition factor receptor (MET) amplification may be associated with the resistance of EGFR-mutant lung tumors to TKIs [61,62]. Upon B-Raf kinase (BRAF) inhibition, RTK ligands, including hepatocyte growth factor (HGF), are able to trigger the activation of the mitogen-activated protein kinase (MAPK) pathway and induce drug resistance [63].

Although researchers have invested a lot of effort to overcome drug resistance [64,65,66,67], the challenges persist, necessitating further in-depth investigations.

#### 2.2.2. Toxicity and Adverse Reactions

Initially, TKIs were considered an emerging treatment modality with lower toxicity than chemotherapy and radiotherapy. However, TKIs have also been found to cause some specific and non-specific side effects during their clinical application. The toxicity of TKIs in clinical use depends on their properties, dosage, and combination with other drugs, as well as patient characteristics and cancer characteristics. The toxicity of TKIs can result from target toxicity, which is the over-inhibition of specific kinases in signaling pathways [68]. This over-inhibition affects not only tumor cells but also the function of normal cells. At the same time, the relatively limited specificity of TKIs leads to off-targeting, which is the unexpected binding of TKIs to kinases other than the targeted kinase, resulting in unintended kinase regulatory effects [68]. The occurrence of any of the above scenarios can lead to TKI toxicity [69,70]. TKIs have been observed to cause vascular dysfunction leading to cardiotoxicity [71]. The risk of clinical heart failure associated with TKI therapy appears to be highly associated with agents with anti-vascular endothelial growth factor (VEGF) activity [72,73]. TKIs can also cause dose-dependent pancreatic toxicity during treatment. Cancer patients treated with TKIs often develop gastrointestinal (GI) symptoms [74]. Meanwhile, TKIs have been reported to be associated with various mild to moderate skin reactions in patients, especially epidermal growth factor receptor TKIs and BCR–ABL TKIs [75,76]. Clinically, to overcome the problem of drug resistance during tumor treatment, combining TKIs with other drugs is a common strategy. That is, by using multiple drugs at the same time, which may involve several different mechanisms of action, the tumor is controlled in multiple ways [77]. While attempting to overcome drug resistance, combination therapy strategies also bring new risks and challenges. For example, multi-drug combinations may exacerbate the incidence and severity of adverse effects; there may be overlapping or synergistic toxicities or unknown side effects between these drugs. Trastuzumab emtansine, commonly abbreviated as T-DM1, is an antibody–drug conjugate that plays a pivotal role in targeted cancer therapy. A study of T-DM1 in combination with docetaxel (with or without trastuzumab) for human HER2-positive breast cancer showed that the combination led to more severe adverse events related to skin toxicity than single-agent T-DM1, and 64% of patients with locally-advanced breast cancer even experienced ≥ grade 3 adverse events [78]. Therefore, prevention as well as management of these toxic reactions during treatment with TKIs is essential for improving patients’ quality of life and treatment adherence.

## 3. The Importance of PTPs in Various Diseases

### 3.1. PTPs in Cancers

#### 3.1.1. Tumor-Suppressive PTPs

A large number of protein tyrosine phosphatases (PTPs) are pivotal tumor suppressors, and their dysregulation is frequently implicated in the pathogenesis of diverse malignancies. As mentioned above, tyrosine kinases, including the EGFR, VEGFR, and MET receptors, play significant roles in oncogenic signaling cascades that govern cellular proliferation, survival, and invasion [11,12,13]. PTPs mitigate these oncogenic effects by dephosphorylating critical nodes within these pathways [7]. PTEN, a phosphatase endowed with both lipid and protein phosphatase activities, is among the most commonly inactivated tumor suppressors in cancer [79]. The functional loss of PTEN can activate the oncogenic PI3K/AKT/mTOR signaling axis, rendering it a prototypical target for therapeutic intervention [80]. PTPN12, recognized as a tumor suppressor, has received great attention as a promising therapeutic target. The re-expression of PTPN12 has been demonstrated to attenuate lung metastasis and to impede tumor progression in breast cancer [3]. PTPN12 modulates the activity of the ATP-dependent ubiquitin separase (p97/Vcp), thereby influencing tumor cell growth and invasion through the regulation of Cas phosphorylation [81]. The utility of PTPN12 as a molecular marker for predicting cancer aggressiveness and patient prognosis has been substantiated by multiple studies [82]. Comprehensive genomic analyses, including whole-genome, whole-exome, and targeted sequencing, have identified PTPRB as a critical angiogenesis suppressor gene through its dephosphorylating action on VEGFR and vascular endothelial (VE) cadherin [83,84]. Additionally, the ablation of INPP5J has been reported to expedite breast cancer tumor growth in vivo by activating the oncogenic PI3K/AKT signaling pathway [14].

Notably, both our studies and that of others have indicated that protein tyrosine phosphatase receptor type O (PTPRO) may serve as a therapeutic target across various cancers (Table 1). PTPRO suppresses lymph node metastasis in esophageal carcinoma by dephosphorylating MET at Y1234/1235 [85]. It also regulates the phosphorylation status of HER2 at Y1248, thereby inhibiting HER2-driven breast oncogenesis [86]. In HER2-positive breast cancer, PTPRO deficiency is associated with lapatinib resistance and a poor prognosis [26]. Furthermore, hypermethylation of the PTPRO gene has been proposed as a potential biomarker for early cancer detection [87,88,89,90,91,92]. Emerging evidence suggests that PTPs influence tumor survival and progression by modulating the activation and function of immune cells, including T cells, B cells, natural killer cells, and macrophages [93]. PTPRO gene deletion enhances programmed death-ligand 1 (PD-L1) secretion in monocytes and macrophages via the JAK2/STAT1 and JAK2/STAT3/c-MYC pathways, which results in immunomodulation [94]. Truncated PTPRO has been identified as a potent tumor suppressor that regulates immune cell growth, differentiation, activation, and immune responses, underscoring its potential role in tumor immunotherapy [93,95].

#### 3.1.2. Oncogenic PTPs

Protein tyrosine phosphatases (PTPs) can, paradoxically, function as oncogenes [101]. For example, PTPN2 (also known as TC-PTP) and its paralogue, PTPN1 (also known as PTP1B), are centrally positioned within cancer development and antitumor immunity, with a wide array of cellular functions attributed to them [102]. A plethora of studies have demonstrated that PTPN2 and PTP1B (PTPN2/N1) contribute to the aggressiveness of cancer by modulating key pathways and molecules such as PTEN, Src activation, and the PKM2/AMPK/mTOC1 pathway [15,16]. Remarkably, PTP1B has been identified as an androgen-receptor-regulated phosphatase that fosters neuroendocrine differentiation and progression in prostate cancer [103]. It has been revealed that the deletion of PTPN2 in T cells bolsters the development and activation of cytotoxicity in CD4+ Th1 cells and CD8+ T cells by amplifying JAK/STAT signaling and IFNγ secretion [104,105,106]. PTPN2-null T cells protect Tp53+/− mice from developing tumors and inhibit the growth of implanted breast cancer by producing cytokines and elevating the population of tumor-activated CD4+ and CD8+ effector/memory cells [105]. Moreover, eliminating PTPN2 in tumor cells primes antitumor immunity by sensitizing tumors to IFNγ [105]. Simultaneously, deletion of the T-cell-specific PTP1B promotes antigen-induced CD8+ T-cell expansion and cytotoxicity against solid tumors through enhanced STAT5 signaling [107]. Recently, research has navigated around the highly polar active sites of phosphatases and characterized a novel, orally bioavailable, and potent PTPN2/N1 active-site inhibitor: ABBV-CLS-484 (AC484) (PubChem CID: 155103607) [105]. This inhibitor intensifies JAK-STAT signaling and ameliorates T-cell dysfunction. AC484 is currently undergoing Phase I clinical trials as a standalone treatment and in synergy with programmed death-1 (PD-1) blockade for solid tumors (ClinicalTrials.gov identifier NCT0477794). PTPN2/N1 are thus emerging as intracellular checkpoints curbing T-cell-mediated antitumor immunity [107], positioning PTPN2/N1-targeted therapy at the crux of a dual anti-cancer strategy by directly attacking tumor cells and fortifying the antitumor efficacy of immune cells.

SHP2, encoded by *PTP1B1*, is another PTP that has been extensively investigated in the context of cancer development, mainly because of its facilitation of signaling pathways, such as RAS/RAF/ERK, PI3K/AKT, JAK/STAT, and RTKs, across a variety of cancers [108,109,110,111,112,113,114,115,116,117,118,119]. SHP2 also serves as a vital modulator of the tumor microenvironment. For instance, T-cell activation is restricted by SHP2′s dephosphorylation of the costimulatory molecules CD28 and CD226 [120]. Additionally, SHP2 attenuates IL-2 production mediated by T-cell receptor (TCR) signaling through the dephosphorylation of critical components such as ZAP70 kinase, CD3ζ, PKC-θ, and PLCγ2 [121]. An array of SHP2 inhibitors, such as SHP099, TNO155, and RMC-4550, has been identified, serving as potential adjuncts to bolster antitumor immunity [120]. Moreover, phosphatase of regenerating liver-3 (PRL-3), a dual-specificity, non-receptor PTP, has been shown to be instrumental in promoting the metastatic progression of various cancers [122]. PRL-3 enhances APC/CFZR1 activity by destabilizing AURKA through the dephosphorylation of FZR1, thus impacting the aggressive traits of colorectal cancer [17]. The overexpression of PRL-3 is associated with the hyperactivation of the EGFR signaling cascade in multiple human cancer cell lines [2]. Nonetheless, the precise functions and regulatory mechanisms of PRL-3 within immune cells and the broader immune response remain areas ripe for investigation.

### 3.2. PTPs in the Nervous System

PTPs are involved in regulating a variety of complex neuronal processes, and their role in axon growth, guidance, and regeneration is functionally recognized [123,124]. PTKs and PTPs collaborate to regulate phosphorylation levels in order to maintain homeostasis, which is essential to ensure the proper functioning of signaling cascades that influence neuronal behavior, as well as neuronal growth, differentiation, and function. In mouse models, PTP1B is a key mediator of synaptic dysfunction and memory deficits in Alzheimer’s disease [125]. PTPN2/N1 are involved in central nervous system disorders and induce hypothalamic insulin disorders [126]. Chemotherapy-related cognitive impairment (CRCI), also known as chemo brain, affects many cancer patients undergoing chemotherapy [127]. The emergence of chemo brain may be due to the drug altering the brain’s structure and function, leading to neuropsychological deficits and cognitive impairment [128,129,130]. PTPRO was identified as being highly expressed in adult brain tissue [131,132]. In a mouse CRCI model, we found that hippocampal PTPRO levels decreased with age [18]. Moreover, we found that a low expression of PTPRO was related to the development of CRCI [18]. The region-specific restoration of hippocampal PTPRO ameliorated doxorubicin (DOX)-induced CRCI in PTPRO−/− mice, suggesting that PTPRO is a promising target for therapeutic intervention in CRCI.

### 3.3. PTPs in Cardiovascular and Metabolic Diseases

Protein tyrosine phosphatases (PTPs), particularly PTP1B (also denoted as PTPN1), are extensively related to the occurrence and development of cardiovascular and metabolic diseases (CVMDs), exhibiting broad expression in related tissues such as human umbilical vein endothelial cells (HUVECs), hepatic tissue, skeletal muscle, and adipose tissue [133]. CVMDs represent the leading cause of mortality worldwide, underscoring the critical imperative for the advancement of novel pharmacotherapies. A hallmark of cardiovascular pathologies associated with obesity and diabetes is endothelial dysfunction [134,135]. PTP1B is emerging as a pivotal regulator of endothelial cell homeostasis, with the selective abrogation of endothelial PTP1B conferring protection against diabetes-induced endothelial dysfunction in conduit arteries via the mitigation of endothelial cell apoptosis [136]. Moreover, the silencing of PTP1B has been shown to ameliorate the inflammatory assault and dysfunction induced by oxidized low-density lipoprotein (ox-LDL) in HUVECs, effecting this through the negative regulation of Krüppel-like factor 2 (KLF2) and the subsequent modulation of the AMPK/SIRT1 signaling axis [137]. Additional research has delineated that the CREB/KMT5A complex augments PTP1B expression in vascular endothelial cells, thus exacerbating hyperglycemia-induced pro-inflammatory cytokines, such as IL-1β, TNFα, and IL-6, in patients with diabetic nephropathy (DN) and in the corresponding rat models [19].

Hyperinsulinemia, a precursor symptom in the ontogenesis of type 2 diabetes mellitus (T2DM), is propelled by the transcriptional activation of PTP1B by the early growth response gene-1 (Egr-1), which, in turn, promotes insulin resistance in hepatic tissues [138]. Experimental evidence from murine models illustrates that short-term strength training enhances hepatic insulin sensitivity in obese mice by diminishing PTP1B abundance independently of variations in body weight [139]. Conversely, the depletion of PTPN2/N1 within the hypothalamic nuclei of obese rodents potentiates central leptin and insulin responsiveness, curtails food intake, and amplifies adipose tissue thermogenesis, culminating in the attenuation of obesity and the amelioration of glucose homeostasis [140]. The global and hepatic-specific excision of the gene for low-molecular-weight protein tyrosine phosphatase (LMPTP) confers resistance against high-fat-diet-induced diabetes without impinging on body mass [141]. Investigators have identified a novel small-molecule LMPTP inhibitor that operates via a non-competitive mechanism, engaging a distinct binding locale adjacent to the catalytic pocket, that displayed a remarkable specificity relative to other phosphatases [141]. Complementarily, phytochemicals have also been investigated for their potential role as PTP antagonists in T2DM management. For instance, Ginsenoside F4, which acts as a PTP1B inhibitor, has been demonstrated to significantly improve the hyperglycemic status of db/db mice and to mitigate skeletal muscle insulin resistance [142]. Chebulinic acid (CA), a dual inhibitor of PTPN9 and SHP2, has been shown to enhance glucose uptake through the stimulation of the AMPK pathway, positing it as a putative pharmacological agent for T2DM management [143].

Recent interest has coalesced around PTP1B inhibitors due to their therapeutic potential in the management of CVMDs [144,145]. For example, the PTP1B inhibitor, MSI-1436, has been reported to bolster hepatic insulin sensitivity via the regulation of autophagic processes, endoplasmic reticulum stress, and systemic inflammatory responses in equine metabolic syndrome (EMS) [20,21,22]. Adjunctive therapy combining leptin with a PTP1B inhibitor (DPM-1001) enhanced glucose metabolism in type 1 diabetes (T1D) in wild-type (WT) mice to levels comparable to those of non-diabetic controls, suggesting its viability as an alternative therapeutic strategy for T1D [146]. Although the quest for selective small-molecule inhibitors capable of targeting PTPs is fraught with challenges, it is anticipated that surmounting these obstacles will result in the creation of new pathways in the therapeutic landscape for CVMDs and their complications.

## 4. The Potential of PTPs as Drug Targets

### 4.1. The Challenges and Emerging Opportunities for PTPs in Cancer Therapy Development

Despite the significant advances in TKIs, phosphatases show new promise as therapeutic targets that can complement existing therapeutic strategies. The complexity of PTPs presents not only pharmacological challenges but also opportunities for innovation. The development of RNA drugs, small molecules, and biologics that can regulate phosphatase activity is opening new therapeutic avenues. For example, small activating RNAs (SaRNAs, consisting of 20 to 26 nucleotides)—a class of short, synthetic RNA molecules—can selectively upregulate the expression of target genes [40]. They work by binding to the regulatory regions of genes, specifically the promoters, and inducing their transcription. In PTP treatment, saRNAs can be designed to enhance the expression of PTPs, thereby increasing their activity and potentially restoring the balance of phosphorylation and dephosphorylation in cells. PTP upregulation provides a subtle approach to cell signaling regulation that aims to restore the signaling pathway balance and to complement existing therapies, which represents an important step in the complex dynamics of cancer cell signaling.

### 4.2. PTP-Targeted Therapy to Overcome TKI Resistance

PTPs either directly target key tumor-associated kinases or influence tumor progression by modulating multiple downstream pathways. These properties are crucial for overcoming drug resistance caused by the activation of aberrant signaling pathways or oncogene alterations. The oncogenic and tumor-suppressive properties of PTPs offer at least two strategies for treating cancer: inhibiting oncogenic phosphatases, akin to suppressing oncogene expression, and reactivating or enhancing the expression of tumor suppressor phosphatases, which are often selectively inhibited in tumor tissue. A series of studies on combined kinase inhibitors have demonstrated that these inhibitors induce a redistribution of signals that is phosphorylation-dependent, which is associated with drug resistance [6,7,8,9]. This necessitates considering the future of targeted therapeutics in terms of tumor suppressor phosphatase activation. For example, PTPN12 inhibits the proliferation of triple-negative breast cancer by interacting with various oncogenic tyrosine kinases to inhibit HER2, PDGFR-β, and EGFR RTKs [3]. PTP1B and PTPN2, pivotal phosphatases for ALK, play a crucial role in determining the responsiveness to ALK TKIs in anaplastic large-cell lymphomas through their regulatory actions on ALK and SHP2 phosphorylation levels. The concurrent targeting of SHP2 and ALK significantly boosts the therapeutic effectiveness of ALK inhibitors, overcoming TKI sensitivity and resistance in ALK-positive ALCL cases [23]. PTP1B controls the activation of several receptor tyrosine kinases, including EGFR and MET, as well as the JAK/STAT pathway by dephosphorylating members such as TYK2, JAK2, and STAT5 [23]. PTPN2 regulates the JAK/STAT pathway by dephosphorylating JAK1/3, STAT1, STAT3, and STAT5 [23]. The loss of DUSP4 leads to the abnormal activation of the MAPK pathway and to uncontrolled MEK and JNK pathways, and it involves the downstream ETS-1 and c-JUN transcription factors, playing an important role in the development and progression of basal-like breast cancer [24]. In breast cancer, PTPRO in tumor-derived exosomes regulates tumor cell invasion and migration and is expected to become a therapeutic target [147]. PTPRO significantly affects the immune infiltration of CD8+ T cells and its status helps to predict the immunotherapy response of patients [25]. In HER2-positive breast cancer, PTPRO deficiency is associated with poor prognosis and drug resistance [26].

**Figure 2:**
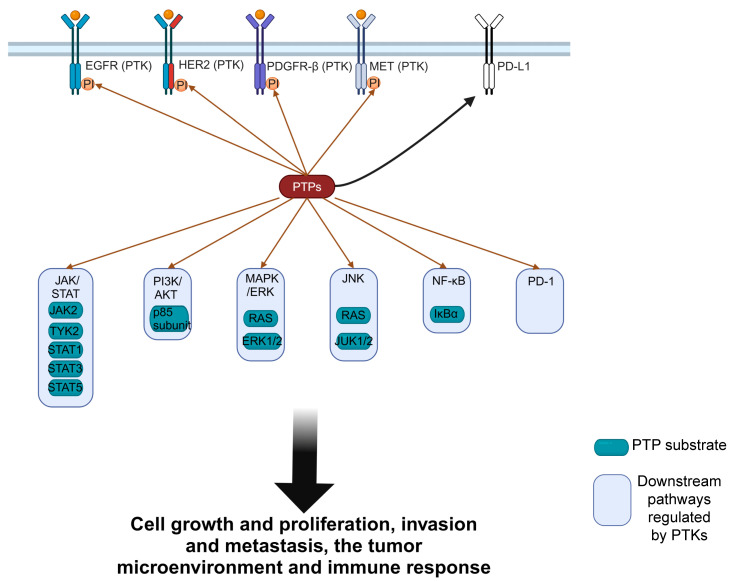
PTPs either directly target PTKs or affect multiple downstream pathways. PTPs can directly dephosphorylate PTKs, such as epidermal growth factor receptor (EGFR), human epidermal growth factor receptor 2 (HER2), and platelet-derived growth factor receptor (PDGFR). Additionally, PTPs can regulate pathways affected by PTKs by dephosphorylating components in the Janus kinase/signal transducer and activator of transcription (JAK/STAT), phosphatidylinositol 3 kinase/protein kinase B (PI3K/AKT), mitogen-activated protein kinase/extracellular signal-regulated kinase (MAPK/ERK), Jun N-terminal kinase (JNK), and nuclear factor-kappa B (NF-κB) pathways. Furthermore, PTPs can regulate programmed death-1 (PD-1)/programmed death-ligand 1 (PD-L1) signaling.

Figure 2 shows that PTPs can directly target PTKs or downstream signaling pathways affected by PTKs. Only the parts mentioned in this review are shown.

### 4.3. Potential Advantages of PTP Inhibitors in Reducing Toxicity

The highly conserved structure of the PTPs’ active sites contributes to a reduced likelihood of off-target toxicity in PTP-targeted therapy [122,148,149]. In MC38 tumor-bearing mice, the PTPN2/PTP1B inhibitor ABBV-CLS-484 shows dose-dependent immune activation and reversible immune infiltration of in vivo tissues that is dependent on its administration status [105]. Similarly, without causing appreciable immunological-related toxicity, compound 182 is a potent and selective competitive inhibitor of the PTP1B and PTPN2 active sites [150]. Additionally, the humanized antibody Prl3-zumab, which targets PRL3, shows minimal side effects in animal models [151,152]. These findings strongly support ongoing investigations into PTP-targeting drug therapies.

Table 2 presents the effects of some of the most recent PTP-targeting drugs and TKI drugs in clinical or preclinical experiments.

## 5. Exploration of PTPs as Drug Targets

Historically, the academic community has been dedicated to the research and development of orthogonal inhibitors of PTPs. However, the high positive charge of the PTP active site results in a preference for negatively charged molecules, while its conservation makes it difficult to design drugs that bind selectively to phosphoryl groups other than those to which PTPs naturally bind. These characteristics limit the cell permeability, bioavailability, and drug discovery potential of the designed drugs [27]. The subsequent generation of potent, selective, and bioavailable PTP inhibitors for therapeutics has been mainly unsuccessful. Therefore, PTPs have been regarded as “undruggable” and “difficult” targets [27,28].

However, the past decade has seen the feasibility of selectively targeting all major classes of phosphatases. The recent successes in this area necessitate a deeper understanding of the structural and regulatory mechanisms of phosphatases to avoid repeating past failures in targeting their catalytic centers. For example, progresses have been seen on non-competitive inhibition, irreversible inhibition, and traditional reversible competitive inhibition techniques continues [27,33,34]. These endeavors have yielded a plethora of high-quality orthosteric structural inhibitors. The emerging trend of the allosteric inhibition of small molecules offers a selective method for inhibiting cell permeability by avoiding charged PTP active sites. For instance, MSI-1436, an allosteric inhibitor of PTP1B, has been shown to positively regulate HER2 signaling in breast cancer development [32]. The most recent PTPN2 and PTP1B active-site inhibitor, ABBV-CLS-484 (AC484), is the first active-site phosphatase inhibitor to enter clinical evaluation for cancer immunotherapy [105]. With AC484, this study provides evidence that cancer immunotherapy with oral PTPN2/N1 systemic inhibitors is well tolerated and effective in multiple preclinical models, including those that are resistant to PD-1 blockade [105]. Additionally, natural or synthetic compounds that inhibit PTPs have been identified, such as PTP1B [161,162,163,164]. The recent success in targeting phosphatases is also attributed to innovative drug development approaches, including nucleic acid drugs for activation or inhibition, as well as compounds that stabilize protein–protein interactions (PPIs) [36,37,38,39,40,41].

Among the strategies outlined in Table 3, nucleic acid drugs offer an alternative approach for modulating gene expression and developing highly specific PTP-targeted drugs. Small interfering RNA (siRNA) technology, as a highly specific antisense therapy for silencing gene expression, has garnered significant attention [38,165]. An approach using small activating RNAs (saRNAs) for sequence-specific gene activation has been proposed, along with tumor suppression by targeting the activation of tumor suppressor PTPs [40]. RNA-targeted therapies can target virtually any genomic component within a cell, including many targets inaccessible to small-molecule drugs and antibodies, providing a natural advantage to overcome the “incurable” challenge. Small-activating-RNA therapies can specifically target certain sites in gene promoters to upregulate expression in an AgO2-dependent manner, thereby restoring the natural function of the protein [166,167]. It is anticipated that the activation of PTPRO by targeting saRNAs will be a promising treatment and prevention strategy for a variety of diseases, including CRCI.

## 6. Navigating the Challenges in PTP-Targeted Drug Development

In this section, we discuss the unresolved queries and particular obstacles in PTP-targeted treatments that must be addressed in order to translate these findings into the clinic.

### 6.1. Challenges for Small-Molecule Drugs

The quest for effective, selective, and bioavailable small-molecule drugs for PTPs is constrained by the highly conserved and positively charged nature of their active sites [176]. It is evident that small-molecule drugs, including allosteric inhibitors, require further enhancement in terms of potency and selectivity. Concurrently, PTPs may employ a multitude of strategies that are contingent upon the enzymes’ distinctive active-site characteristics, structure-related regulatory mechanisms, and pioneering drug development approaches. For instance, bidentate ligands bind to the allosteric and catalytic sites of PTPs, which can improve inhibitor selectivity.

### 6.2. Delivery Challenges for Antibody and Nucleic Acid Drugs

Antibody drugs targeting PTPs may exhibit immune escape due to poor tissue permeability and tumor antigen loss. They may also cause excessive immune activation in the body, leading to serious side effects. Nucleic acid drugs also encounter similar difficulties. Their targeting accuracy and delivery mechanisms are not optimal, resulting in low concentrations of nucleic acid drugs at the target. This necessitates increasing the dose, which may induce associated adverse reactions.

### 6.3. Other Challenges

PTPs have the potential to be developed into selective and bioavailable probes, but this still requires further investigation. For the development of allosteric inhibitors, the isolation of full-length recombinant PTP proteins requires greater efficiency. The development of small-molecule inhibitors is in full swing, but small-molecule activators also require further research.

### 6.4. Integration and Innovation in Drug Development

These observations suggest that further investment in the field of PTP-targeted treatment is warranted. In the context of the current era of artificial intelligence, the development of more predictive models and the participation of machine learning/artificial intelligence methods in drug design represent an emerging field of research [177]. Artificial intelligence may provide significant assistance in the development of ortho-inhibitory drugs that target the active site of PTPs. Once the challenges of drug design are overcome, a significant number of patients may benefit from these emerging drugs.

## 7. Conclusions

Since their entry into the clinic, TKI tumor-targeted drugs have greatly changed treatment approaches. TKIs were rapidly developed due to their advantages in drug delivery, specificity, and pharmacokinetic/pharmacodynamic models. These advantages greatly promoted the development of TKI-targeted therapy and made revolutionary changes to existing targeted therapies. However, at the same time, a series of unanticipated challenges arose.

The traditional approach of targeting TKI oncogenes needs to be further improved, which also forces us to think about the future of tumor treatment from another perspective—targeting tumor suppressors. Kinases and phosphatases, as two sides of a coin, participate in various cellular functions, and their balance plays an important role in the human body. We have discussed the significance of protein phosphatases. PTPs play a pivotal role in oncology and other medical fields, including immunity, neurology, and metabolism.

PTPs, due to their oncogenic and tumor-suppressive characteristics, provide at least two cancer treatment strategies. The strategy of targeting oncogenic protein tyrosine phosphatases (PTPs) aligns with the classical approach to oncogene therapy. As this strategy is rapidly advancing, the concept of “undruggable” is changing to “difficult” and may perhaps even change to “good patentable drug targets” in the future. However, anti-cancer therapeutic efforts focused on activating or enhancing tumor-inhibiting PTPs aim to restore their physiological function and stability [6,7,8,9,178,179]. This approach enables us to consider and expand the arsenal of anti-cancer therapies from the perspective of activating or enhancing tumor inhibition. Consequently, we underscore the substantial potential of identifying and employing PTPs to combine the inhibition of cancer-promoting PTPs with the restoration of cancer-suppressing PTPs, which could significantly alter the landscape of cancer treatment. A growing understanding of the biological and structural functions of PTPs suggests that enzymes previously considered untreatable may in fact be viable targets for a range of human diseases. Over the next decade, it is anticipated that several PTP-targeted drugs will be granted approval by the FDA as oncology therapeutics. Additionally, in other areas of disease treatment, PTP-targeted drugs will become attractive as well.

## Figures and Tables

**Table 1:** Downstream effectors of protein tyrosine phosphatase receptor type O (PTPRO) in human cancer, which mediate multiple tumor-related downstream targets.

Effectors	Functions	Tumor Types	References
HER2 (immediate)	PTPRO inhibited HER2-driven breast cancer through dephosphorylation, leading to dual effects of HER2.	Breast cancer	[86]
SYK (immediate)	Inhibited cell proliferation. Induced apoptosis.	Lymphoma Diffuse large B-cell lymphomas	[96,97]
MET (immediate)	PTPRO could inhibit MET-mediated metastasis.	Esophageal squamous cell carcinoma (ESCC)	[85]
AKT serine/threonine kinase (AKT)/mammalian target of rapamycin (mTOR) (immediate)	PTPRO silencing induced the activation of the AKT serine/threonine kinase (AKT)/mammalian target of rapamycin (mTOR) signaling axis.	Colorectal cancer	[98]
TLR4	Inhibited cell proliferation.	Hepatocellular carcinoma	[99]
P53/FOXM1 (immediate)	Inhibited the leukemic cell population. Suppressed inflammatory cells in the spleen.	Chronic lymphocytic leukemia	[100]
NFkB	Inhibited cell proliferation.	Hepatocellular carcinoma	[99]
Oncogenic fusion protein BCR–ABL	Inhibited cell growth. Enhanced drug-induced apoptosis. Reduced tumorigenic potential.	Chronic myelogenous leukemia	[100]

SYK, spleen tyrosine kinase; TLR4, Toll-like receptor 4.

**Table 2:** The efficacy of PTP drugs and tyrosine kinase inhibitors (TKIs) in clinical or preclinical trials.

Compound	Target	Stage of Development	Preclinical Study Results *	Refs.
TNO155	SHP2 (PTP)	Phase I clinical trial (NCT03114319)	TNO155 showed benefit in four out of six mouse PDX models.	[153,154]
RMC-4630	SHP2 (PTP)	Phase I clinical trial (NCT04916236) and Phase I/II clinical trial (NCT03989115)	RMC-4630 accelerated and increased the magnitude of the regression of ocitinib-sensitive EGFR-mutant tumors in mice.	[155]
PRL3-zumab	PRL3 (PTP)	Phase II clinical trials (NCT04118114, NCT04452955)	PRL3-zumab showed efficacy in the treatment of nude mouse PRL-3+ SNU-484 orthotopic tumors.	[156]
ABBV-CLS-484	PTPN2/PTP1B (PTP)	Phase I clinical trial (NCT04777994)	AC484 treatment induced highly significant tumor regression and improved survival in all four mouse models.	[105]
JBJ-04-125-02	Fourth-generation EGFR TKI (PTK)	Preclinical	JBJ-04-125-02 reduced the minimum residual tumor size by an average of nearly 60 mm^3^ in H1975 xenograft mice.	[157]
BLU-945	Fourth-generation EGFR TKI (PTK)	Phase I/II clinical trial (NCT04862780)	BLU-945 strongly inhibited tumor growth in both NCI-H1975 mouse models.	[158]
U3-1402	HER3 Fourth-generation EGFR TKI (PTK)	Phase III clinical trial (NCT05338970)	In the human MDA-MB-453 breast cancer cell line xenograft model, U3-1402 treatment led to significant tumor regression with a TGI of 87%.	[159]
BBT-176	Fourth-generation EGFR TKI (PTK)	Phase I/II clinical trial (NCT04820023; terminated)	In all three EGFR-mutant PDX models, BBT-176 showed dose-dependent tumor suppression.	[160]

* As clinical trial results for some new drugs are not yet available, we only present the results of preclinical studies here.

**Table 3:** Approaches to PTP-related drug design currently being studied.

Drug Design Method	Function and Gene	Mechanism or Manner	Reference
Small-molecule targeting agent	Activates PTEN	Natural compounds inhibit WWP1 and reactivate PTEN	Indole-3-carbinol [168]
	Activates SHP-1	SHP-1 reactivation by multiple protein kinase inhibitors	Regorafenib [29]
	Suppresses SHP2	Active-site inhibitors	Cryptotanshinone and II-B08 [30,31]
		Allosteric inhibitor	SHP099 [30,35]
	Suppresses PTP1B	Reversible and non-competitive inhibitor	MSI-1436 [32]
	Suppresses PTP1B, PTPN2	Active-site inhibitors	ABBV-CLS-484 (AC484); compound 182 [105,150]
Antibody drug	PRL3	Humanized antibody	PRL3-zumab [152]
	HER2	HER2-directed antibody–drug conjugates (ADCs)	Trastuzumab emtansine, trastuzumab deruxtecan [169]
Genome editing	Knocks out *PTPN6*	CRISPR-Cas9	[170]
	Knocks out *PTPN2*	CRISPR-Cas9	[171]
Nucleic acid drugs	PTPN22	siRNA; PTPN22 silencing	[36]
	SHP-1	siRNA; SHP-1 reactivation by RNF38 silencing	[37]
	PTPN13	siRNA-mediated PTPN13 silencing	[38]
	/	small activating RNAs (saRNAs)	[40]
Epigenetic modifications	/	DNA methylation; histone methylation and acetylation	[172]
Other	Suppresses TC-PTP	Proteolysis-targeting chimera (PROTAC)	[39]
	PTEN	PIK3R1 positively regulates PTEN activity in combination with PTEN (protein–protein interaction (PPI))	[41]
	PTEN	Phosphorylation of Tyr336 by FAK increases the phosphatase activity of PTEN	[173,174,175]

## Data Availability

No data was used for the research described in the article.

## Abbreviations

PTKs, protein tyrosine kinases; TKs, tyrosine kinases; PTPs, protein tyrosine phosphatases; ATP, adenosine triphosphate; TKIs, tyrosine kinase inhibitors; BRCA1, breast and ovarian cancer susceptibility gene 1; PARP, poly (ADP-ribose) polymerase; RTK, receptor tyrosine kinase; DUSPs, dual-specific phosphatases; nRTKs, non-receptor tyrosine kinases; EGFR, epidermal growth factor receptor; PI3K, phosphatidylinositol 3 kinase; MAPK, mitogen-activated protein kinase; FDA, Food and Drug Administration; PK/PD, pharmacokinetic/pharmacodynamic; NSCLC, non-small-cell lung cancer; KRAS, Kirsten rat sarcoma viral oncogene homolog; BRAF, B-Raf proto-oncogene serine/threonine kinase; HGF, hepatocyte growth factor; VEGF, vascular endothelial growth factor; HER2, human epidermal growth factor receptor 2; MET, mesenchymal–epithelial transition factor receptor; PTPRB, protein tyrosine phosphatase receptor type B; VEGF, vascular endothelial growth factor; INPP5J, inositol polyphosphate 5-phosphatase PIPP; AKT/PKB, protein kinase B; PTEN, phosphatase and tensin homolog; PTPRO, protein tyrosine phosphatase receptor O gene; SHP2, src-homology 2-containing protein tyrosine phosphatase 2; JNK, c-Jun N-terminal kinase; JAK, Janus kinase; TYK2, tyrosine kinase 2; PTPN2, protein tyrosine phosphatase non-receptor type 2; PTPN1, protein tyrosine phosphatase non-receptor type 1, PTP1B; TC-PTP, T-cell protein tyrosine phosphatase; SHP1/*PTPN6*, src-homology region 2 domain-containing phosphatase-1; PTPA, protein tyrosine phosphatase A; PTP-1B, protein tyrosine phosphatase 1B; CRCI, chemotherapy-related cognitive impairment; PRL3, phosphatase of regenerating liver 3; FAK, focal adhesion kinase; PPI, protein–protein interaction; SaRNAs, small activating RNAs.
